# Left Atrial Appendage: Mere Spectator or Protagonist? A Narrative Review of Its Multimodal Imaging Assessment and Its Closure Techniques

**DOI:** 10.3390/jcm15187230

**Published:** 2026-09-17

**Authors:** Martina Belli, Gianpiero Italiano, Francesca Lassandro Pepe, Francesca Vespasiano, Roberto Celotto, Raffaele Scaffa, Andrea Salica, Paolo Ciancarella, Tanya Salvatore, Donatella Cantisani, Tiziana Salatino, Ruggero De Paulis, Fabrizio Tomai

**Affiliations:** 1 Division of Cardiology, Department of Cardiovascular Sciences, European Hospital, 00149 Rome, Italy; 2 Department of Cardiovascular Sciences, Aurelia Hospital, 00165 Rome, Italy; 3 Department of Cardiac Surgery, European Hospital, Unicamillus University, 00131 Rome, Italy

**Keywords:** left atrial appendage, left atrium, multimodality imaging, atrial fibrillation, left atrial appendage occlusion

## Abstract

**Background:** For years, the left atrial appendage (LAA) was considered a simple recess of the atrial wall, but recently it has gained its own respectability in the cardiovascular field, therefore its accurate assessment cannot be overlooked in clinical practice. Percutaneous or surgical closure of the LAA has become a valid and safe alternative to reduce the risk of stroke in patients with non-valvular atrial fibrillation (AF), who have long-term contraindications to anticoagulant therapy. **Results:** This progression was feasible thanks to the developments and the improvements of devices, surgical and imaging techniques. **Objectives**: This review explores the essential role of multimodality imaging in LAA, including new imaging tools, and its impact on the optimization of closure techniques and outcomes. **Conclusions**: Multimodality imaging is mandatory for each step along the therapeutic path: patient selection, pre-procedural planning, procedural monitoring, and follow-up.

## 1. Introduction

### LAA Anatomy

The left atrial appendage (LAA) derives from the primordial left atrium (LA), which is formed mainly by the absorption of the primordial pulmonary veins and their branches. It is a finger-like projection from the main body of the LA. The junction is fairly well defined by a narrowing at the orifice of the appendage. There are considerable variations in its size, shape, and relationship with adjacent cardiac and extracardiac structures, which can be extremely relevant when interventional procedures are performed. In most hearts, the LAA extends between the anterior and the lateral walls of the LA, and its tip is directed anterosuperiorly, overlapping the left border of the right ventricular (RV) outflow tract or the pulmonary trunk and the main stem of the left coronary or the circumflex artery. It is not uncommon to find the tip of the LAA directed laterally and backward. However, in a small percent of hearts, the tip of the LAA passes behind the arterial pedicle to sit in the transverse pericardial sinus. The external appearance of the LAA is that of a slightly flattened tubular structure with crenellations, often with one or more bends and terminating in a pointed tip. Because of its slightly flattened shape, the lower surface usually overlies the left ventricle (LV) and the upper surface is beneath the fibrous pericardium. Internally, the orifice of the LAA is usually oval, whereas round, triangular, and water-drop shapes are observed less frequently. The left lateral ridge separates the orifices of the left pulmonary veins from the LAA orifice, but the precise relationship between the level of the orifice and its distance to the venous orifices varies. The smooth muscular wall of the LA vestibule separates the orifice from the mitral annulus. Most appendages have a well-defined orifice that leads to a neck region that opens to the body of the appendage. In a large study of postmortem hearts, Veinot et al. defined lobes as protrusions from the main body with the tail portion also representing a lobe, whereas bends in the tail do not constitute more lobes. They found that two lobes were most common (54%), followed by three lobes (23%), one lobe (20%), and four lobes (3%), and noted there were no significant age- or sex-related differences in LAA morphologies. An increased number of lobes was associated with the presence of a thrombus independent of clinical risk and blood stasis [1,2]. In a study using multidetector computed tomography (MDCT) and cardiac magnetic resonance (CMR), the shapes of the LAA in patients with drug-refractory AF were classified into four morphological types, with “chicken wing” being the most common (48%), followed by “cactus” (30%), “windsock” (19%), and “cauliflower” (3%). “Cauliflower” morphology—the least frequent type—showed the highest numerical association with embolic events [2], though this finding is limited by the small subgroup size and the recognized reproducibility challenges of LAA morphology classification, and should be interpreted as hypothesis-generating rather than definitive. Normal functioning of the LAA reduces the risk of thrombus formation [3]. In fact, during sinus rhythm, physiological contractility ensures adequate blood flow, preventing stasis [4].

## 2. Multimodality Imaging

### 2.1. Echocardiography

**Figure 1:**
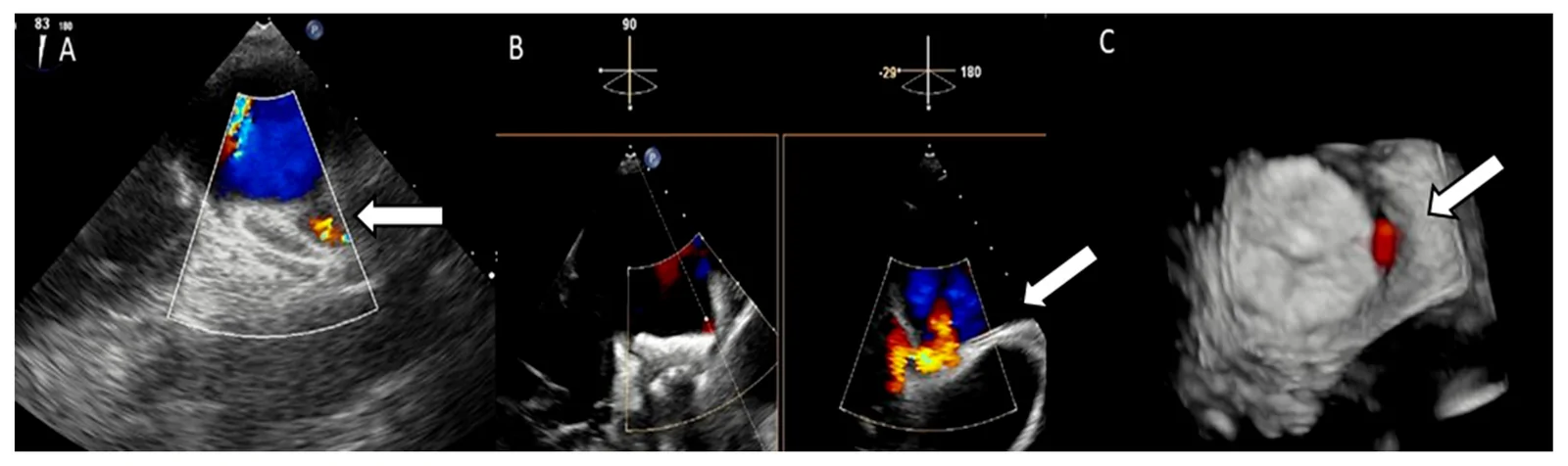
A colorDoppler interrogation of the LAA device (**A**). TEE demonstration of the device in two orthogonal planes showing a residual peri-device leakage (PDL) (**B**). A 3D color Doppler en face view of the device demarcating the crescent-shaped nature of the leakage (**C**).

**Figure 2:**
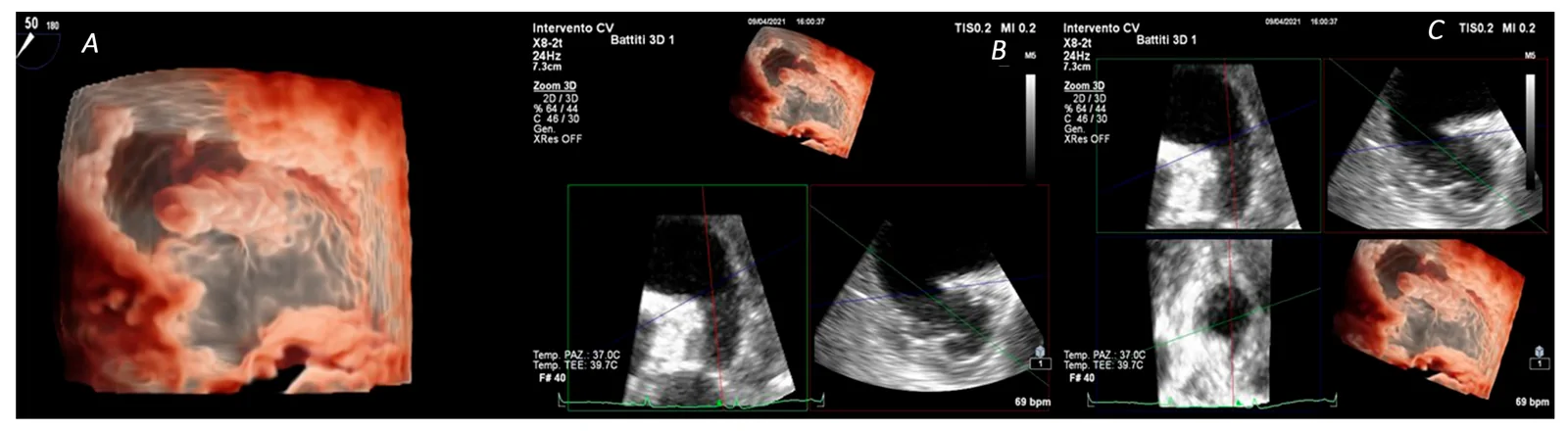
A new 3D TEE rendering modality with improved transparency (Glass) (**A**) and 3D TEE with multiplanar reconstruction allow for accurate and quick assessment of LAA morphology and dimensions (**B**,**C**).

**Figure 3:**
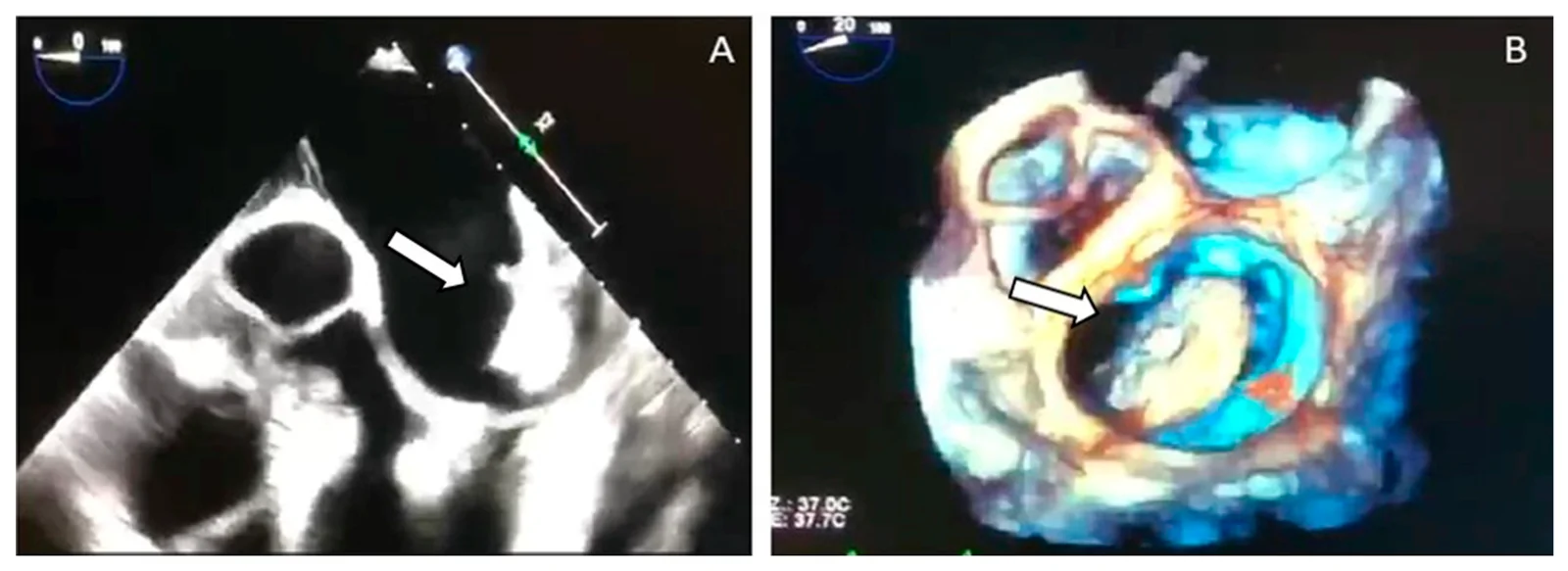
Two-dimensional, zoomed (**A**), and three-dimensional (**B**) TEE show embolization of an LAAC device into the left atrium (LA).

Transesophageal echocardiogram (TEE) is the principal imaging modality for the structural and functional evaluation of the LAA [5]. It produces high-resolution images that are essential for determining the size of the ostium and landing zone, ruling out thrombi, analyzing the complex anatomy of the LAA and providing real-time guidance [6]. TEE assessment obtains a comprehensive morphological evaluation that may be used for implantation planning, device selection and size and potential complication prediction [7]. The standard 2D TEE protocol involves positioning the probe in the mid-esophagus and performing an imaging sweep from 0° to 135°, often with significant counterclockwise rotation. The pre-procedural TEE determines patient eligibility and informs the overall procedural strategy [8,9]. Real-time TEE guidance is fundamental for a safe and appropriate transeptal puncture, typically in an inferior/posterior location of the interatrial septum (IAS) [10]. This location ensures the most favorable sheath orientation for the device, whereas a puncture site too superior and anterior carries a risk of perforating the aortic root [11]. The development of three-dimensional (3D) TEE has transformed LAA assessment by providing greater anatomical and functional insights [12]. A crucial aspect of 3D-TEE is the “en face” view, which provides a direct perspective of the LAA ostium from the LA. This view is invaluable for a correct assessment of the orifice’s morphology, which can help to predict the risk of peri-device leaks (Figure 1). It also effectively delineates the relationship between the ostium and adjacent structures like the mitral valve (MV), left pulmonary veins, and the interposed ridge. The 3D multiplanar reconstruction (MPR) method is considered the most precise for measurements of the orifice, landing zone dimensions, and LAA depth during echocardiographic assessment. Critically, measurements derived from 3D-TEE have been proven to be more reproducible and to correlate more closely with the actual device size used for closure [13]. In addition, 3D MPR imaging is crucial for a precise assessment of the LAA’s morphology and for thrombus detection. The use of advanced rendering technologies in 3D-TEE has greatly improved the morphological assessment of the LAA [14]. These innovative methods produce high-definition, realistic 3D reconstructions of the LAA by manipulating light sources and transillumination (such as True-Vue and Glass-Vue) (Figure 2 and Figure 3) [15]. These realistic images help operators quickly and intuitively understand the LAA’s anatomy. Moreover, combining a modality like Glass-Vue with automated 3D measurement software, LAA evaluation has been simplified, leading to a faster and more accurate anatomical assessment. Intracardiac echocardiography (ICE) has been proposed as a valuable alternative to TEE, especially in patients with contraindications for general anesthesia or esophageal intubation. Several studies have confirmed its safety and efficacy while also reporting reductions in procedural time and fluoroscopy dose [16]. However, the use of ICE presents some limitations. It requires dedicated venous access, which can increase procedural complexity. Data from a large registry revealed a higher incidence of pericardial effusions requiring intervention at 45 days in the ICE cohort compared to the TEE group. While not statistically significant, major adverse events were also numerically more frequent in ICE-guided procedures [17]. Despite these challenges and the high cost of single-use catheters, ICE facilitates conscious sedation and, importantly, in registry data, has shown no statistically significant difference in peri-device leak rates compared with TEE-guided procedures [17]. However, these comparisons derive from unadjusted observational registries subject to confounding by indication, and the numerically higher rates of pericardial effusion and major adverse events in ICE-guided cohorts [16] preclude definitive equivalence claims pending prospective comparative data. Functional assessment of the LAA complements morphological evaluation and is integral to pre-procedural workup. TEE enables measurement of LAA emptying velocities—normally exceeding 0.2 m/s in sinus rhythm, with lower values indicating impaired contractility and hemostatic risk. Spontaneous echo contrast (SEC) is graded from 1 (mild swirling) to 4 (dense sludge), reflecting progressive blood stasis and escalating thromboembolic risk; the presence of sludge or dense SEC, even without a discrete thrombus, should influence pre-procedural decision-making and may affect device selection. LAA phasic strain, assessable by speckle-tracking TEE, provides quantitative insight into appendage mechanical function and correlates with hemostatic indices, though its role in routine clinical practice remains investigational [4]. While transthoracic echocardiography (TTE) offers limited direct LAA visualization due to acoustic window constraints, it contributes to initial LA functional characterization, gross SEC detection in selected patients, and screening for LA thrombus when TEE access is not immediately available [4]. Integrated imaging platforms are transforming procedural guidance for LAAC. Echocardiography/fluoroscopy fusion systems enable real-time TEE anatomical overlay on fluoroscopic imaging, reducing contrast dependency and improving spatial orientation during transseptal puncture and device deployment. CT-fluoroscopy roadmapping projects pre-procedural volumetric anatomy onto live fluoroscopy, providing a three-dimensional reference frame that facilitates device alignment and reduces procedural time. Automated segmentation algorithms trained on CT and TEE datasets enable rapid volumetric LAA reconstruction and dimensional measurement, reducing operator variability; AI-guided ICE has been proposed as a further tool to standardize positioning and alert operators to suboptimal device configurations [17]. These systems exemplify a broader transition in medical imaging AI, away from narrow, single-purpose algorithms and toward generalist foundation models capable of multi-task anatomical analysis across diverse imaging contexts [18], with direct implications for structural heart disease workflows. As prospective validation data accumulate, this integration is expected to advance procedural reproducibility and outcome standardization.

### 2.2. MDCT and CMR

**Figure 4:**
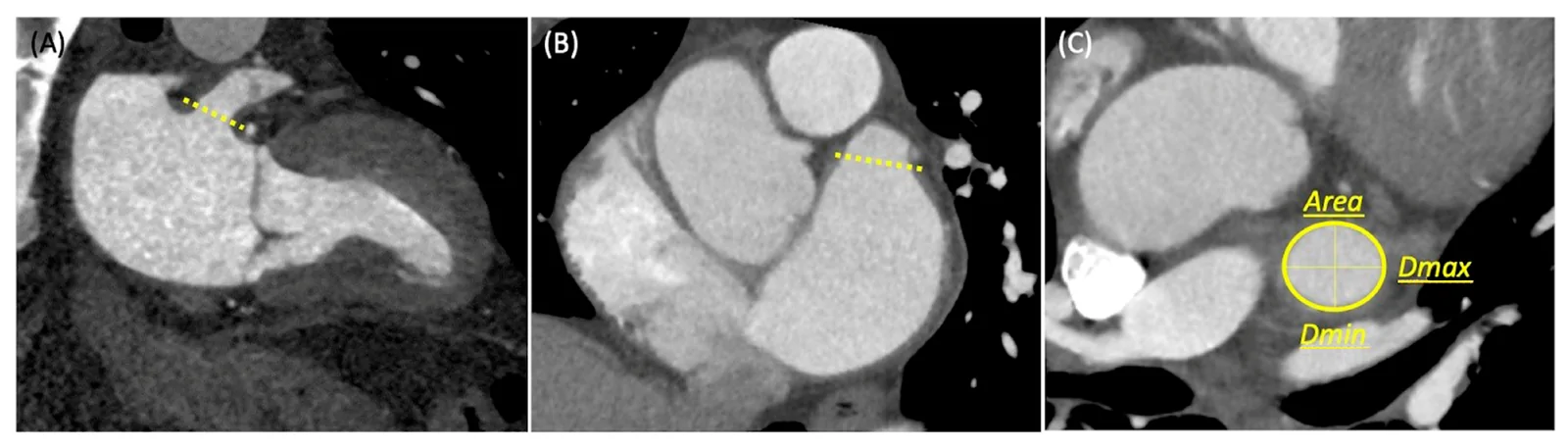
Standard CT assessment of the LAA: correct identification of the LAA ostium on two orthogonal planes of the left atrium in end-systolic images (dotted lines, panels (**A**,**B**)); measurement of ostial diameters and planimetric area for device sizing in an oblique cross-sectional plane (panel (**C**)).

**Figure 5:**
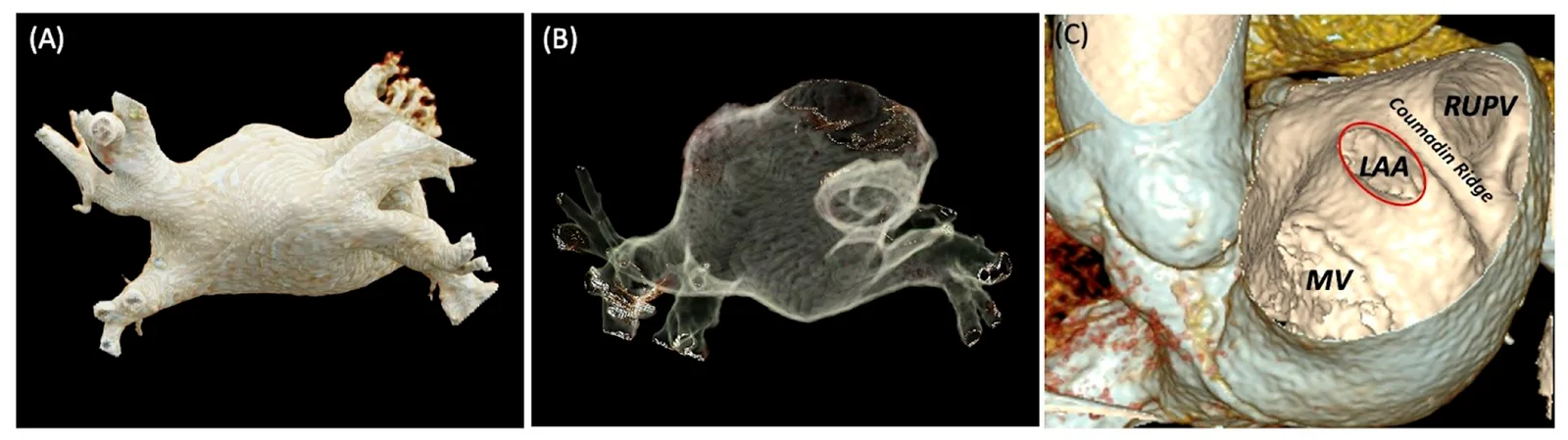
Advanced CT assessment of the LAA: 3D cinematic rendering (panel (**A**)) and translucent reconstruction (panel (**B**)) easily depict anatomy of the left atrium and pulmonary veins; intracavitary reconstruction better defines the relationship of the LAA ostium with surrounding structures (panel (**C**)). MV = mitral valve; LAA = left atrial appendage; RUPV = right upper pulmonary vein.

**Figure 6:**
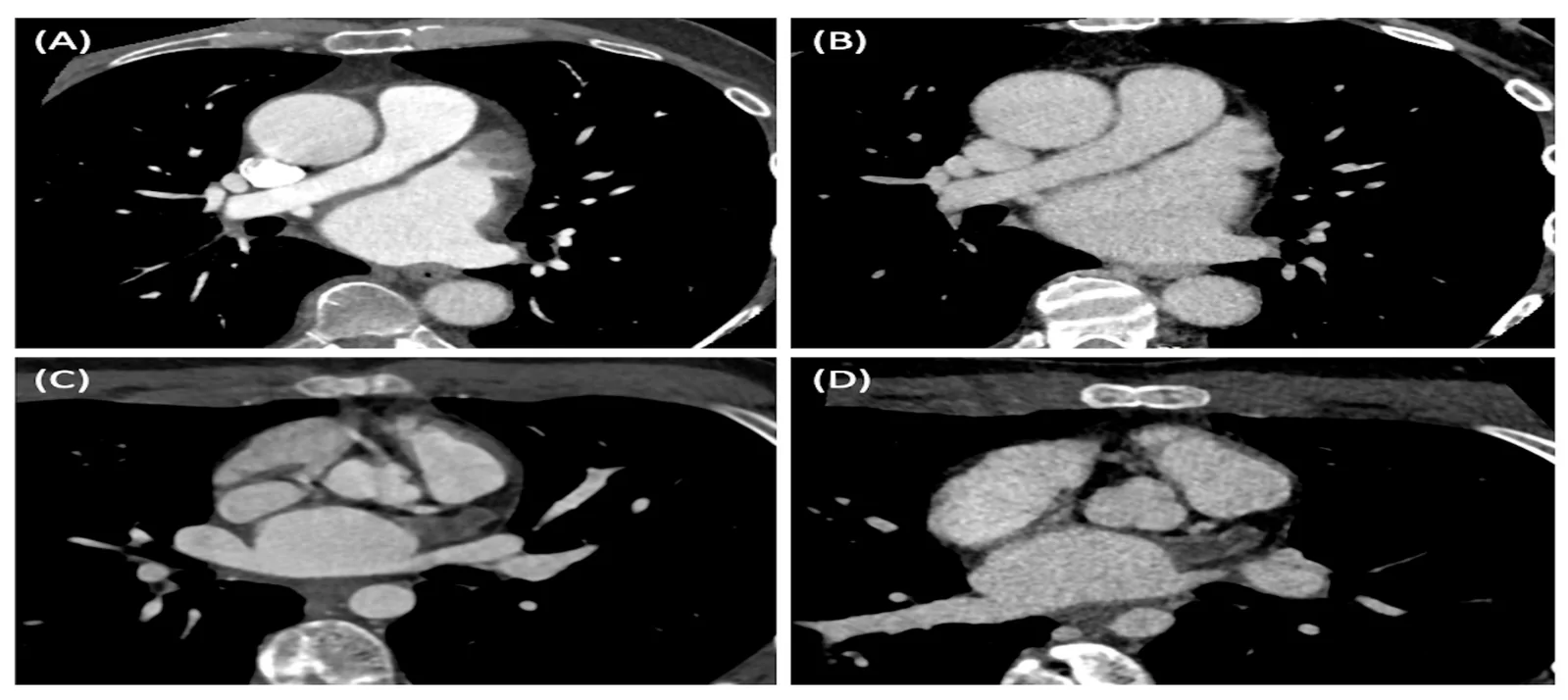
Thrombus rule-out with CT: angiographic phase showing a suspected filling defect of the LAA (panel (**A**)) and delayed phase of the same patient demonstrating normal opacification of the left appendage (panel (**B**)); other angiographic-phase image showing a suspected LAA thrombus (panel (**C**)) with corresponding delayed-phase image confirming the presence of thrombus within the LAA (panel (**D**)).

**Figure 7:**
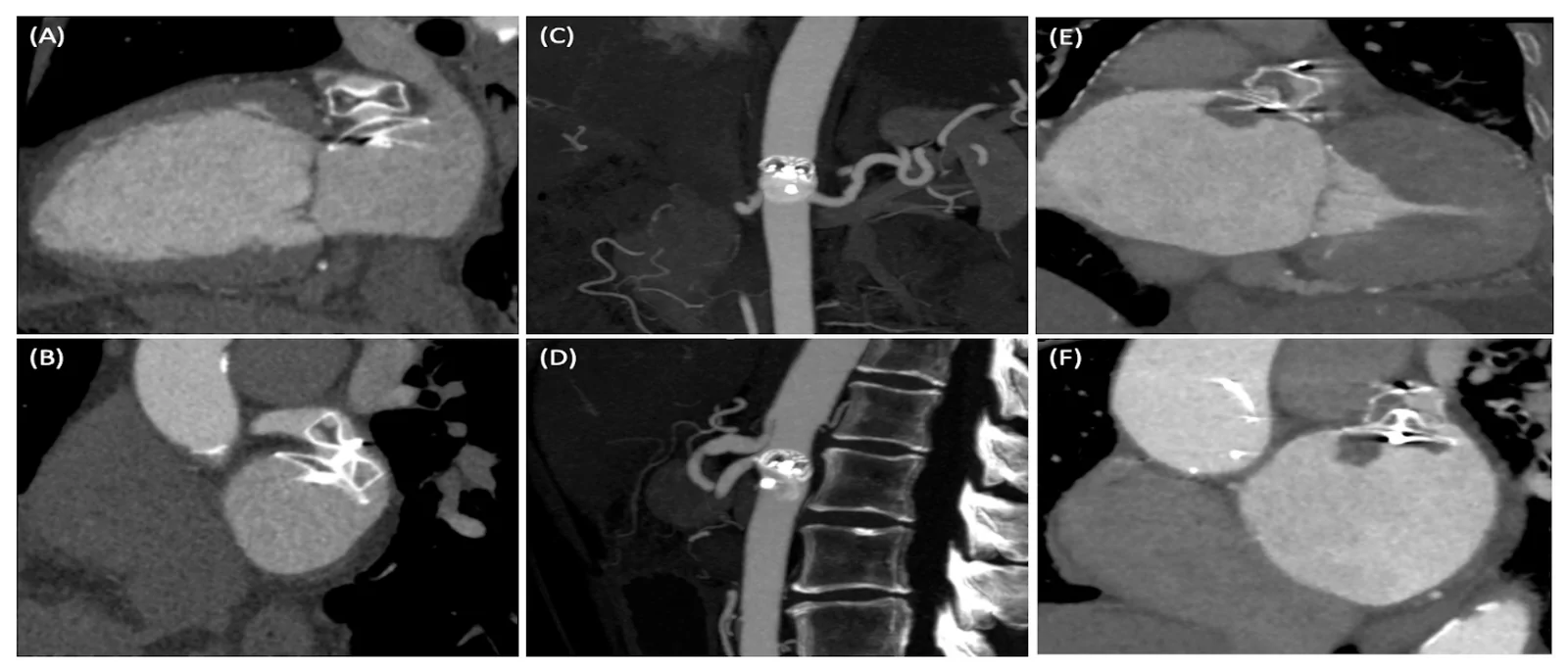
Post-procedural complications after LAAC: dislodgement and incomplete endothelization of the device with residual LAA patency (panels (**A**,**B**)); device embolization in the abdominal aorta (panels (**C**,**D**)); device-related thrombus (panels (**E**,**F**)).

Although TEE plays a key role and remains the most commonly used imaging strategy for LAA evaluation, left atrial appendage closure (LAAC) planning relies on a multimodal imaging approach. In this setting, MDCT represents a non-invasive alternative imaging modality, which does not require sedation. The main strengths of MDCT are its high spatial resolution and favorable temporal resolution, a wide field of view that is not constrained by body habitus or acoustic window limitations, lower dependence on the operator skill, and the ability to acquire a volumetric image dataset for multiplanar reconstructions. Pre-procedural MDCT should be performed with the objective of determining the anatomic feasibility of the LAAC procedure, to provide accurate sizing of the LAA dimensions for device selection, peri-procedural planning, and exclusion of LAA thrombus. A multidetector CT scanner with at least 64 detector rows is required for an adequate-quality scan. CT scanning is typically performed with ECG-gated retrospective acquisition to visualize the LAA anatomy across the cardiac cycle. Nevertheless, prospective ECG-gating in systole is also feasible for this purpose [19]. From a safety perspective, dose optimization strategies (low-iodine protocols, iterative reconstruction, and artifact reduction), aligned with ALARA (As Low As Reasonably Achievable) principles, are increasingly integrated into practice and effectively balance diagnostic yield with protection of renal function and radiation exposure. With isotropic voxel sizes, MDCT enables precise delineation of the LAA ostium, landing zone, and the overall morphology (windsock, chicken wing, cactus, cauliflower, and other variants), which are essential for device selection, sizing, and deployment strategy. Post-processing multiplanar reconstructions are used to obtain orthogonal views of the neck of the LAA and thus a double-oblique en face view of the orifice when it is largest, typically in the ventricular end-systolic phase (30–40% R-R interval), for measurement of maximal and minimal diameters, perimeter, and area. Long-axis views easily allow identification of the landing zone—the optimal deployment plane for the device—which varies across device models according to the manufacturers’ specifications, and enable measurement of the dominant lobe length, a parameter essential for device sizing [20] (Figure 4). In most centers, device sizing is based on an integrated analysis of measurement obtained with 2D modalities and MDCT. Echocardiographic measurements are typically smaller than those obtained with CT. When measurements are discordant, the larger diameters are adopted to avoid undersizing and related complications. In this setting, CT-based device selection proves to be a more accurate method than conventional 2D-TEE-based sizing [21,22]. In the study by Wang et al., patients planned with MDCT had a 100% rate of successful implantation, compared to the 88% success rate of patients planned with TEE in the PROTECT AF trial [23]. In addition, CT offers a comprehensive assessment of surrounding structures (interatrial septum, Coumadin ridge, pulmonary veins, left circumflex artery and great cardiac vein, coronary bypass grafts, etc.) and potential anomalous access routes that may influence access strategy and post-implant stability (Figure 5). In the pre-procedural assessment, it is also essential to exclude the presence of thrombus in the LAA, which represents a contraindication to the closure procedure in centers lacking extensive expertise. The ideal CT protocol comprises a two-phase acquisition with a delayed scan, which allows distinguishing endoluminal thrombus stratification from a pseudo-filling defect due to slow flow with high accuracy (Figure 6). When compared to TEE, combined arterial and delayed-phase CT demonstrated high diagnostic performance for the detection of LAA thrombus, with a sensitivity of 100%, specificity of 99%, positive predictive value of 92%, and negative predictive value of 100%. These findings support the role of CT as a reliable alternative to TEE for LAA thrombus assessment in appropriate clinical settings [24,25]. A pivotal adjunct to CT-based planning is 3D printing. CT-derived, patient-specific 3D-printed models of the LAA provide tangible visualization of contour, lumen diameter, and the spatial relationship between ostium and lobes, enabling pre-procedural device testing and sizing simulations. Moreover, 3D-printed models have been shown to correlate with intra-procedural device sizing, reducing device waste and potentially shortening procedural time. CT also plays a central role in post-procedural surveillance after LAAC. Following implantation, CT provides high-resolution assessment of device position and complete sealing of the LAA, and is highly effective at detecting peri-device leaks, device-related thrombus and device dislocation/embolization. Multiphase acquisitions, including a delayed phase, enhance sensitivity for residual patency and subtle leaks that may escape detection on other imaging modalities. When compared with transesophageal echocardiography, CT offers superior delineation of peri-device anatomy and endothelialization of the device surface, particularly in complex morphologies [26]. CT also permits a comprehensive evaluation of other thoracic structures and enables rapid identification of acute complications such as pericardial effusion, major bleeding, or embolic events, guiding timely intervention (Figure 7). Cardiac MR may represent a promising alternative modality for planning and follow-up of LAAC, but at present its role remains limited to selected cases, especially when iodinated contrast use or radiation exposure are contraindicated. MRI enables pre-procedural planning through CT-like sizing concepts, using 3D ECG-gated sequences to visualize the anatomy of the LAA (ostium, depth, landing zone) and morphology [27,28] (Table 1). MR-based sizing approaches have demonstrated feasibility in prospective cohorts, achieving high implantation success and accurate device sizing, though broader adoption requires validation, resource availability, and standardized protocols in routine practice.

## 3. LAA Exclusion/Occlusion

### 3.1. Surgical Approach

In recent guidelines, surgical closure of the LAA is recommended as a Class I indication in patients with AF undergoing concomitant valve surgery, LAA closure representing one of the main pillars in the treatment of AF in order to reduce cardioembolic events [29,30,31]. The larger clinical trial LAAOS III concluded that in patients with AF who had undergone cardiac surgery the risk of ischemic stroke was lower with concomitant LAA closure than without it, and its advantages appear to be additive to oral anticoagulation therapy (OAC) [32]. From a surgical point of view, appendage closure can be performed via standard median sternotomy or minimally invasive techniques depending on surgeon preferences and expertise. Several surgical procedures are available for LAA closure. Two main approaches are described in the surgical armamentarium represented by excision and exclusion techniques. The excision approach comprehends stapler resection and conventional LAA excision and suture. These techniques are characterized by the excision of the LAA and suture of the residual stump at the base of LAA from its external face. This approach has an intrinsic complexity due to an adjunctive cardiotomy in the setting of the main cardiac procedure. Moreover, the inherent fragility of the LAA wall makes these strategies challenging and potentially dangerous in terms of risk bleeding. Furthermore, due to the different anatomical types in terms of the macroscopic morphology of the LAA, this procedure is not always fully standardizable and may result in incomplete closure. Surgical exclusion approaches are represented by external ligation, internal purse-string ligation or by closure with dedicated epicardial clipping devices; external exclusion by the use of dedicated clip devices evidenced better results compared to the other approaches [33,34]. Although operative LAA closure does not significantly increase operative time and is not associated with increased rates of complications such as bleeding or prolonged hospitalization [35], closure techniques remain a matter of debate due to the rate of incomplete or inadequate closure during follow-up [36,37]. Moreover, unsuccessful LAA closure represented by a patent LAA excluded LAA with persistent flow into the appendage, a residual stump that was greater than 10 mm in the LAA in maximum length, and may worsen the risk of thromboembolic events [38,39].

### 3.2. Percutaneous Approach

**Figure 8:**
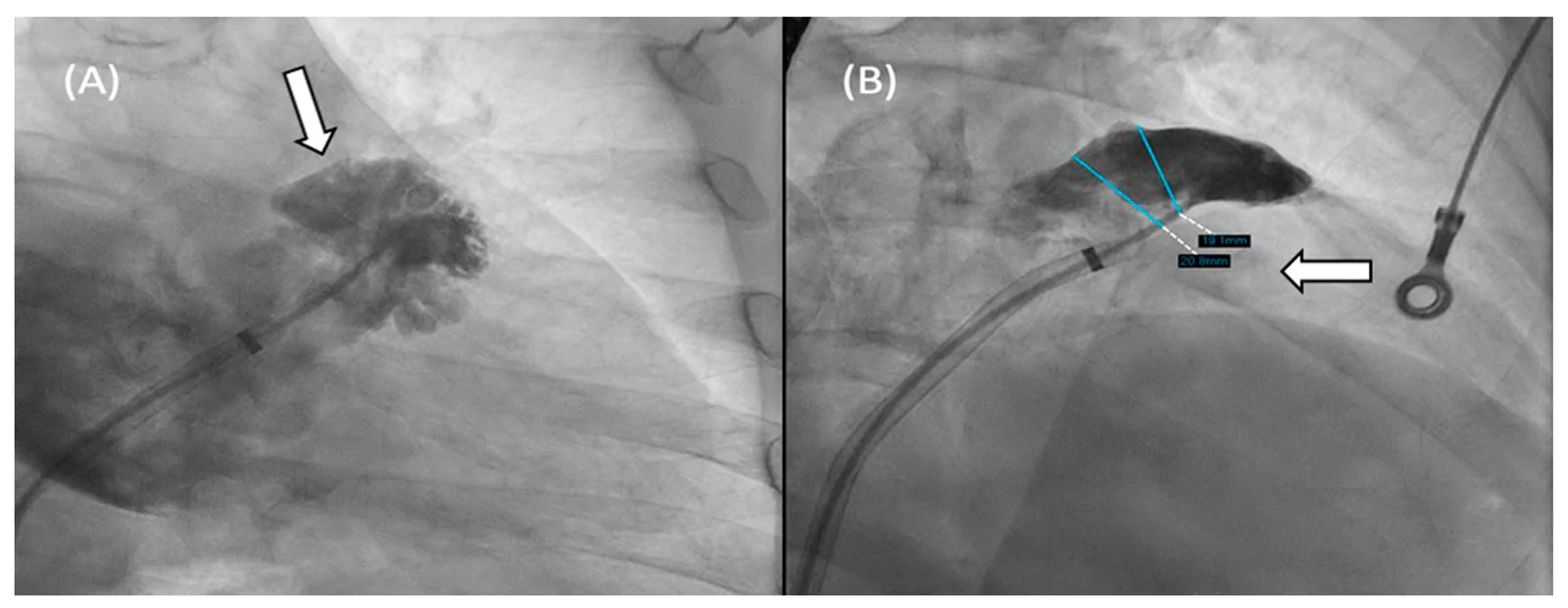
Angiography showing the morphology of left atrial appendage (Panel (**A**)); measurements for the correct implantation of the device (Panel (**B**)).

**Figure 9:**
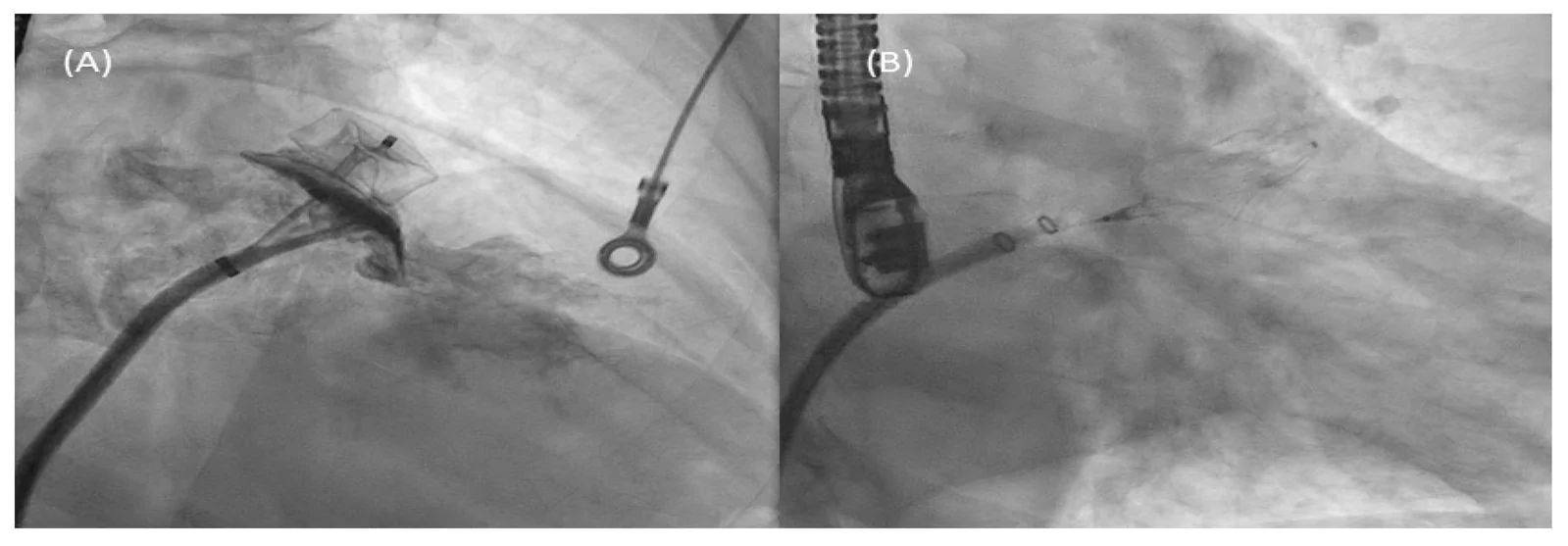
Complete sealing of the LAA before release of the Amulet device (Panel (**A**)); tug test before final release of Watchman device (Panel (**B**)).

Percutaneous left atrial appendage occlusion (LAAO) is preferably performed via the right femoral vein using ultrasound-guided micropuncture to reduce access-site complications [40]. The transseptal puncture (TSP) is tailored to pre-procedural TEE and computed tomography angiography (CTA) and then verified intra-procedurally. A posterior—typically mid-to-inferior—TSP creates a posterior-to-anterior vector coaxial with the anteriorly directed LAA, improving sheath maneuverability and working length while reducing aortic contact. Anatomic variants (e.g., chicken-wing or reverse chicken-wing) may require minor adjustments (slightly more anterior/central or more inferior) to achieve a straighter trajectory [41]. Septal crossing may be achieved with needle, wire, or radiofrequency. After positioning of the transseptal sheath, a pigtail catheter is advanced in the LAA to minimize trauma; then fluoroscopic and echocardiographic measurements are obtained to confirm ostial diameter, depth, angulation, and lobar anatomy [11] (Figure 8). The delivery sheath is then advanced coaxially into the LAA and a step-by-step procedure is performed according to the utilized device. For single-lobe plugs, the PASS framework is applied—Position, Anchor, Size (target compression), and Seal—followed by a standardized tug test and leak assessment (≤5 mm, as accepted in trials) before release (Figure 9). When seating, anchoring, compression (~10–30%), and sealing are satisfactory, the delivery cable is detached and the sheath withdrawn into the right atrium [42]. Device evolution began with PLAATO (a nitinol frame covered with expanded polytetrafluoroethylene), which showed feasibility but was discontinued for commercial reasons [43]. The Amplatzer platform introduced the lobe-and-disc design, subsequently refined in Amulet, improving anchoring and sealing with high implant success and favorable echocardiographic/radiographic profiles. In parallel, Watchman 2.5 provided the first randomized evidence versus warfarin (PROTECT-AF, PREVAIL) [44,45], demonstrating non-inferiority and, over time, reductions in major and intracranial bleeding; large United States registries confirmed safety in routine practice. The Watchman FLX iteration addressed mechanical limitations of 2.5 (recapturability, compression range, perforation risk, peri-device leak), lowering complications, embolization, and residual leaks [46]; it is the current U.S. reference and shares European prominence with Amulet [47]. Additional systems include LAmbre (conforming central joint), WaveCrest (single-lobe, multiple anchors), Ultraseal (bulb-and-sail), and CLAAS (polyurethane foam-lined endoskeleton), several of which are still under evaluation. LARIAT [48] excludes the LAA epicardially (dual access) and, despite conceptual appeal, has reported leak/thrombosis signals that limit routine use [46]. Safety has improved with operator experience and device refinement, yet three adverse events remain central: pericardial tamponade (typically within 6–24 h; ~1.3% with Watchman 2.5 [49]; 1.0% PINNACLE-FLX and 0.51% SURPASS with Watchman FLX; 2.4% with Amulet vs. 1.2% with Watchman in AMULET IDE [47]), device embolization (~0.07% Watchman 2.5; 0–0.1% FLX; ~0.7% Amulet [47]), and periprocedural stroke (<0.5%), most often due to air embolism [50] or de novo thrombosis with subtherapeutic activated clotting time (ACT) [42] (Table 2). Peri-device leak (PDL), even small, raises stroke/systemic embolism risk; CT outperforms TEE for detection/mechanism and guides secondary closure with vascular plugs, coils, or radiofrequency ablation when indicated [51].

## 4. Discussion

For years, OAC has been the standard of care in preventing thromboembolic events, initially with vitamin K antagonist (VKA) [56] and currently with direct oral anticoagulants (DOACs) [57] with a class I indication for AF thromboembolic prevention to all eligible patients according to the new CHA2DS2-VA score [55]. In this context, age and sex have long be debated regarding their specific weight in the overall patient assessment.

Sex influences the risk of stroke, its subtype, and the severity of outcomes [58]. Furthermore, it was shown that female patients with AF and ischemic stroke were older, experienced higher stroke severity, and had worse outcomes; despite this, adjustment for age, pre-stroke functional status, and baseline severity attenuated the excess mortality [59]. Female sex was for a long time incorporated into thromboembolic risk stratification. Over the years, and following several clinical studies, evidence has shown that female sex is an age-dependent risk modifier rather than an independent risk factor per se [60].

In terms of age, data from the PREFER in AF [61] and ANAFIE registries [62] have shown that patients aged ≥85 years have a higher incidence of stroke/systemic embolism compared with younger elderly patients. However, in this age group, thromboembolic risk is not determined by age alone, but is further modulated by previous stroke, AF pattern, renal dysfunction, blood pressure, diabetes-related factors, and other clinical characteristics. Even more importantly, the absolute benefit of anticoagulation increases in very elderly patients, as their higher thromboembolic risk translates into a greater absolute number of preventable events. Therefore, in light of all these considerations, the current European guidelines on AF have reaffirmed the importance of age in CV risk stratification, while reconsidering female sex not as an independent risk factor per se, but rather as an age-dependent risk modifier. Accordingly, the guidelines retain 2 points for age and remove female sex from the risk calculation, moving from CHA_2_DS_2_-VASc to CHA_2_DS_2_-VA [55]. It is important to identify not only the factors that increase the risk of cardioembolism but also those that enable early prognostic stratification in patients with cardioembolic stroke. In particular, an observational prognostic study showed that early recurrent embolization, impaired level of consciousness, motor deficits, heart failure, advanced age, and male sex were independently associated with an increased risk of in-hospital mortality [63]. Although OAC significantly reduces thromboembolic events in patients with AF, it does not completely eliminate the risk [64]. In this scenario, both surgical and percutaneous LAAC have become particularly attractive. However, achieving procedural success and the desired outcomes first requires adequate pre-procedural planning, which involves providing an accurate anatomical description of LAA itself, by means of multimodality imaging, mainly through TEE and MDCT and/or CMRI. Studies, in fact, demonstrated that CMRI and MDCT were comparable for LAA sizing and thrombus assessment prior to LAAC the absence of OAC [65]. Subsequently, complete LAA exclusion must be ensured [66] by intra- and post-procedural imaging guaranteeing no residual communication between LAA and LA [32,66,67]. This can be made possible in the context of the multimodality imaging era, in which multiple tools can be used either as alternatives in cases of contraindications (for example, using MRI in case of iodinated contrast media’s contraindication for renal failure) or unavailability of certain modalities, or with the aim of reinforcing the findings obtained from the initial imaging techniques and with the integration of the emerging artificial intelligence [68]. LAAC can be achieved also percutaneously and the Amplatzer Amulet (Abbott, Plymouth, MN, USA) and Watchman (Boston Scientific, Marlborough, MA, USA) devices are most widely used. The principal findings of meta-analysis evaluating stroke prevention strategies among patients with non-valvular AF [69] were a similar efficacy to prevent thromboembolic events between LAAC and OACs, with a numerical (but not statistically significant) higher risk of stroke in the case of LAAC compared to OACs and lower rate of major bleeding (excluding procedural bleeding events) and hemorrhagic stroke for LAAC compared to OACs [70,71,72]. In an RCT including patients with severe aortic stenosis (AS) and AF undergoing transcatheter aortic valve replacement (TAVR), concomitant transcatheter LAAC was non-inferior to medical therapy with respect to a composite primary endpoint including all-cause mortality, stroke, and major bleeding at 2 years [73]. LAA is not merely an anatomic remnant: 1—it contributes to the reservoir function of the left atrium; 2—its contractile activity enables ventricular filling; 3—it is an important source of atrial natriuretic peptide (ANP), contributing to cardiovascular and volume homeostasis; 4—it is a potential source of ectopic activity and arrhythmogenic triggers, particularly in patients with persistent or long-standing persistent atrial fibrillation [74]. In patients with non-valvular AF, the LAA has already undergone pathological fibrotic remodeling, which drastically reduces its native natriuretic and mechanical efficiency [75]. Therefore, considering that AF per se affects the endocrine and mechanical function of the left atrial appendage, LAAC reduces thromboembolic risk without further compromising endocrine or hemodynamic properties [76]. Thanks to these findings, percutaneous LAAC earned a Class IIb recommendation in the current European Guidelines in patients with contraindications for long-term anticoagulant treatment to prevent ischemic stroke and thromboembolism [55]. As a result of the intense research in this field and the recent upgrade in guidelines, it is plausible that procedural safety will be enhanced, treatment personalized, and clinical indications for LAAC consequently expanded. This will likely be driven by the technological development of devices, increasingly anatomical adaptability together with advances in surgical techniques, multimodality imaging and artificial intelligence.

## 5. Conclusions

Surgical or percutaneous exclusion of the LAA from systemic circulation in patients with AF has become a key reference in the scientific field, strengthening its credibility both as an alternative or as a complementary strategy to reduce the risk and severity of stroke and systemic thromboembolism. Although further comparative studies are needed before percutaneous LAAC can be routinely recommended as an alternative to anticoagulation, the available evidence supports its role in patients with contraindications to long-term OAC. In conclusion, multimodality imaging is therefore mandatory for each step along the therapeutic path: patient selection, pre-procedural planning, procedural monitoring, and follow-up.

## Figures and Tables

**Table 1:** Comparison of imaging modalities for LAA assessment in the context of LAA closure.

	2D/3D TEE	MDCT	CMR	ICE
**Spatial resolution**	High	Very high	Moderate–High	Moderate
**Temporal resolution**	High	Moderate (ECG-gated)	Moderate	High
**Thrombus exclusion**	Good (sensitivity ~95%, specificity ~99% with 3D MPR) [9,24,25]	Excellent: two-phase protocol with delayed scan	Good; avoids iodinated contrast—suitable in CKD if gadolinium also avoided or gadolinium-free protocol applied [27,28]	Moderate; limited FOV
**LAA morphology assessment**	Good (3D MPR, Glass-Vue, True-Vue)	Excellent (volumetric, isotropic)	Good (3D ECG-gated sequences)	Limited
**Ostial sizing accuracy**	Good; tends to underestimate vs. CT	Gold standard for device sizing [21,22,23]	Comparable to CT in feasibility cohorts	Adequate for procedural use
**Real-time procedural guidance**	Excellent	Not applicable	Not applicable	Good
**Post-procedural surveillance**	Good for PDL (color Doppler)	Superior: detects subtle leaks, device-related thrombus	Limited; device compatibility issues (improving with newer MR-conditional devices)	Not routinely used post procedure
**Sedation/anesthesia**	Yes (GA or deep sedation)	No	No	No GA required; conscious sedation used
**Radiation exposure**	None	Yes (dose-optimized protocols)	None	None
**Iodinated contrast**	No	Yes	No	No
**Operator dependence**	High	Moderate	Moderate	High
**Availability**	Widely available	Widely available	Limited in many centers	Growing (specialized centers)
**Cost**	Moderate	Moderate	High	High (single-use catheters)
**Key limitations**	Esophageal contraindications; underestimates LAA dimensions; GA-dependent	Radiation; contrast nephrotoxicity; not real-time	Long acquisition times; device compatibility; limited standardization; gadolinium-based contrast contraindicated in severe CKD (eGFR < 30 mL/min/1.73 m^2^); gadolinium-free protocols feasible but less validated	Higher pericardial effusion rates in registry data [17]; access-site complexity; limited FOV
**Role in LAAO pathway**	Pre-procedural planning + intra-procedural guidance (gold standard) [6,10,12]	Pre-procedural sizing + thrombus exclusion + post-procedural follow-up	Selected patients (CKD, contrast allergy, radiation concern)	Alternative to TEE in GA-contraindicated patients

Abbreviations: TEE = transesophageal echocardiography; MDCT = multidetector computed tomography; CMR = cardiac magnetic resonance; ICE = intracardiac echocardiography; LAA = left atrial appendage; LAAO = left atrial appendage occlusion; PDL = peri-device leak; CKD = chronic kidney disease; GA = general anesthesia; MPR = multiplanar reconstruction; FOV = field of view.

**Table 2:** Comparison of major percutaneous LAA occlusion devices.

	Watchman 2.5	Watchman FLX	Amplatzer Amulet	LAmbre	WaveCrest
**Manufacturer**	Boston Scientific	Boston Scientific	Abbott	Lifetech Scientific	Johnson & Johnson/Biosense Webster
**Design**	Single-lobe nitinol plug; fixation barbs; polyethylene terephthalate membrane on LA face	Single-lobe; rounded atraumatic distal end; wider compression range; 18 anchors; fully recapturable	Lobe-and-disc; proximal disc covers ostium independently of lobe position	Central waist + outer frame; conforming to variable ostial shapes	Single-lobe; polyurethane foam seal; multiple anchors
**Available sizes**	21–33 mm	20–35 mm	Lobe 16–34 mm; Disc 22–40 mm	16–36 mm	22, 27, 32 mm
**Key pivotal trials**	PROTECT-AF [44]; PREVAIL [45]	PINNACLE-FLX [52];	AMULET IDE [47]	TREND [53];	WAVECREST I
**Implant success rate**	~88% [44]; ~95% [45]	~98.8% [52]	~98.4% [47]	~97–99% [53]	
**Pericardial tamponade**	~1.3% [48]	~1.0% [52]; 0.51% [54]	~2.4% [47]	~0.5–1.0% [53]	
**Device embolization**	~0.07% [48]	~0–0.1% [52]	~0.7% [47]	<1% [53]	Rare
**Peri-device leak—reported rates (threshold and timepoint vary by trial)**	~32% (any PDL, all sizes) at 12 months by TEE [44] ~11.8% PDL > 3 mm at 12 months [44]	~5% (PDL ≤5 mm) at 12 months [52]	~6.5% at 45 days [47]	~3–8% (series-dependent; definition varies) [53]	
**Recapturability**	Partial	Full (up to 3×)	Partial	Partial	Full
**Periprocedural stroke**	<0.5% [44]	<0.5% [52]	<0.5% [47]	Rare [53]	
**Regulatory/guideline status**	Class IIb—ESC 2024 AF Guidelines (procedure-level) [55] CE marked; FDA approved	Class IIb—ESC 2024 [55] Current US (FDA) and EU (CE) reference device	Class IIb—ESC 2024 [55] CE marked; current EU reference	CE marked (Europe, 2016) [53] NMPA approved (China) Limited availability in other regions	CE marked (Europe, 2013) [48] US regulatory status: no FDA approval
**Key advantage**	First RCT evidence vs. warfarin	Reduced complications vs. 2.5; broadest compression range; full recapture	Independent disc sealing; favorable for complex morphologies	Conforming design for irregular ostia	Foam-enhanced sealing; multiple anchors
**Key limitation**	Higher PDL rates; limited recapturability	Cost; learning curve	Higher tamponade vs. FLX in AMULET IDE; larger disc may complicate retrieval	Limited long-term RCT data	Limited comparative evidence

PDL row: rates are trial-specific and not directly comparable across devices. Only AMULET IDE constitutes a head-to-head randomized comparison. All other figures derive from single-arm trials or registries with differing designs, populations, and endpoint definitions. ESC Class IIb applies to percutaneous LAAC as a procedure in patients with contraindications to long-term OAC; guidelines do not assign separate recommendation classes to individual devices. Abbreviations: LAA = left atrial appendage; LAAC = left atrial appendage closure; OAC = oral anticoagulation; PDL = peri-device leak; RCT = randomized controlled trial; ESC = European Society of Cardiology; FDA = Food and Drug Administration; CE = Conformité Européenne; NMPA = National Medical Products Administration; TEE = transesophageal echocardiography.

## Data Availability

No new data were created or analyzed in this study. Data sharing is not applicable to this article.

## Abbreviations

LAA	left atrial appendage
LA	left atrium
RV	right ventricular
LV	left ventricle
MDCT	multidetector computed tomography
CMR	cardiac magnetic resonance
TEE	transesophageal echocardiogram
IAS	interatrial septum
MPR	multiplanar reconstruction
ICE	intracardiac echocardiography
LAAC	left atrial appendage closure
LAAO	left atrial appendage occlusion
DOACs	direct oral anticoagulants
